# Longitudinally Extensive Transverse Myelitis in a Lupus–Neuromyelitis Optica Overlap

**DOI:** 10.5041/RMMJ.10429

**Published:** 2021-01-19

**Authors:** Yonit Tavor, Moshe Herskovitz, Galia Ronen, Alexandra Balbir-Gurman

**Affiliations:** 1 B. Shine Rheumatology Unit, Rambam Health Care Campus, Haifa, Israel; 2 The Ruth & Bruce Rappaport Faculty of Medicine, Technion–Israel Institute of Technology, Haifa, Israel; 3 Department of Neurology, Rambam Health Care Campus, Haifa, Israel; 4 Department of Radiology, Rambam Health Care Campus, Haifa, Israel

**Keywords:** Aquaporin 4, LETM, NMO, overlap syndrome, SLE

## Abstract

Transverse myelitis is an inflammatory lesion of the spinal cord, occurring in different autoimmune, infectious, and traumatic diseases but is the hallmark of neuromyelitis optica (NMO), a rare neurologic autoimmune disease. Patients with systemic lupus erythematosus (SLE) may develop transverse myelitis as a neuropsychiatric complication of active disease; however, at times, NMO co-exists as an additional primary autoimmune condition in a SLE patient. Correct diagnosis of a SLE–NMO overlap is important not only for the different disease course and prognosis compared with SLE-related LETM, but especially for the emerging and highly specific NMO treatment options, not established for SLE-related LETM—such as anti-aquaporin 4 antibodies, anti-VEGF antibodies, complement modulation, or IVIg.

## INTRODUCTION

Transverse myelitis is an inflammatory lesion of the spinal cord, causing significant morbidity and disability. It occurs in different autoimmune, infectious, and traumatic diseases but is the hallmark of neuromyelitis optica (NMO), a rare neurologic autoimmune disease. Patients with systemic lupus erythematosus (SLE) may develop transverse myelitis as a neuropsychiatric complication of active disease; however, at times, NMO co-exists as an additional primary autoimmune condition in a SLE patient. As the disease course, prognosis, and treatment options differ between these scenarios, it is highly important to acknowledge the possible overlap between these entities. We present a case of relapsing NMO in a patient with SLE (a SLE–NMO overlap) and review the literature.

## CASE PRESENTATION

Our case was a 51-year-old SLE patient, diagnosed 20 years earlier with polyarthritis, Raynaud’s phenomenon, immune thrombocytopenic purpura, and positive immunologic studies including antinuclear (ANA), anti-dsDNA, anti-SS-A antibodies, and low complement levels. She was treated with hydroxychloroquine and steroids which were tapered, and she remained in long-term remission for years.

In 2009 she was hospitalized for acute appearance of left-hand paresis with hypoesthesia. Physical examination revealed distal weakness 4/5, hypoesthesia and astereognosis of her left hand, and positive Romberg test, with no symptoms or signs of SLE activity.

Laboratory tests including complete blood count, liver and kidney function tests, and thyroid hormone levels were all normal; erythrocyte sedimentation rate was 69 mm/hour, whereas C-reactive protein was not elevated. Immune profile revealed positive ANA, anti-dsDNA, SS-A, and SS-B antibodies tests, and no anti-Smith antibodies. Antiphospholipid antibodies (APLA) including lupus anticoagulant, B2 glycoprotein I, and anti-cardiolipin were negative. On lumbar puncture, opening pressure was normal; spinal fluid was clear, with no leucocytes or abnormal cells; glucose was within normal range, protein was 57 mg/dL, and oligo-clonal bands were absent. Carotid artery Doppler ultrasound and transesophageal echocardiography were unremarkable. Retinal examination revealed no signs of vasculitis. Magnetic resonance imaging (MRI) of the brain and cervical spine demonstrated a hyperintense T2 white matter lesion with partial T1 contrast enhancement and no diffusion restriction in the right parietal lobe. Lupus-related involvement of the central nervous system (CNS) was suggested, and the patient was treated with intravenous (i.v.) pulses of methylprednisolone, followed by high-dose prednisone and subsequent taper, along with hydroxychloroquine. The patient improved rapidly, as did her brain MRI.

**Figure 1:**
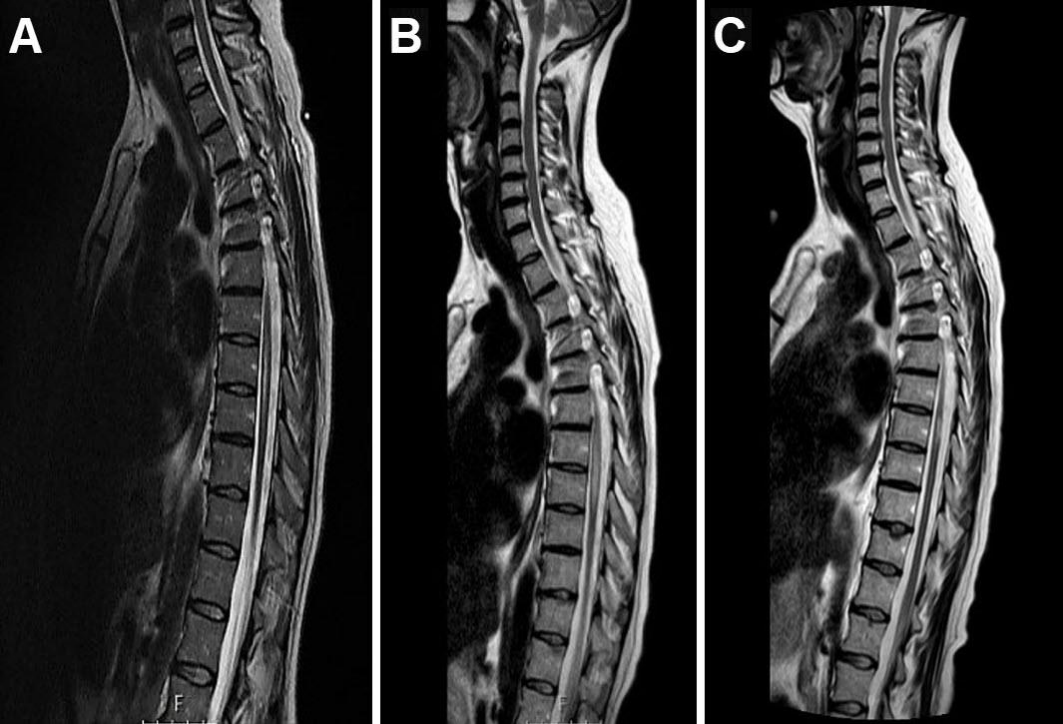
During Prednisone Taper, the Patient Presented with Sensory Loss in Both Legs. Neurological examination demonstrated a D7 sensory level. Spinal MRI revealed an inflammatory longitudinal myelitis lesion extending from D7 to D11, here shown on sagittal T2 of the dorsal spine **(A)**. The patient was treated with induction therapy followed by monthly pulsed i.v. cyclophosphamide infusions. After the 5th infusion, the patient developed para-paresis, urinary incontinence, and sensory level above her legs. A spinal MRI demonstrated a new longitudinal lesion extending D6–D9 **(B)**. Neuro-ophthalmologic studies were negative. Anti-aquaporin 4 antibodies (AQP4) were negative at that time. Induction therapy was re-instituted; maintenance with azathioprine and high-dose IVIg was initiated. The patient’s condition stabilized, and she remained with minimal left-hand paresis and mild spinal ataxia and sensory loss, with improvement visible on repeat MRI **(C)**.

Eight months later, as prednisone dose reached 20 mg/d, the patient was re-admitted to the neurology department for severe sensory loss in both legs. Neurological examination demonstrated a D7 sensory level. Repeated immune-serology was similar to her first admission. Spinal MRI revealed a longitudinal white matter lesion extending from D7 to D11 with a high signal on T2 images compatible with myelitis (Figure 1A). She was treated with i.v. pulses of methylprednisolone, and plasma exchange; induction treatment with monthly 1 g i.v. cyclophosphamide (CYC) infusions was introduced. After the 5th CYC infusion she developed severe neurologic deterioration presenting with para-paresis, urinary incontinence, and sensory level above her legs. Spinal MRI demonstrated a new longitudinal transverse myelitis lesion extending from D6 to D9 (Figure 1B).

No SLE activity, in terms of skin, joints, serous and mucous membranes, kidney, and other systems, was demonstrated in any of her myelitis-related episodes, her dsDNA decreased to become insignificant, complement levels remained normal, and her APLA profile was negative. In search of NMO criteria, neuro-ophthalmologic studies were negative, as were anti-aquaporin 4 antibodies (AQP4). The patient was treated again with pulses of methylprednisolone and plasma exchange sessions; CYC was replaced with azathioprine 150 mg/day, and repeated courses of i.v. immunoglobulin (IVIg) were added (0.4 g/kg/d for 5 consecutive days every month). The patient’s condition stabilized, and an MRI showed improvement (Figure 1C). After rehabilitation she had minimal residual left-hand weakness due to her old cerebral involvement, with mild spinal ataxia and sensory loss, and could return to work as a clerk. By that time, AQP4 were positive on several occasions, starting 2012. Every attempt to space between IVIg pulses resulted in another exacerbation. Another episode of severe para-paresis, urinary retention, and sensory level at D8 developed soon after discontinuation of treatment by the patient (due to incompliance) but responded well to an additional course of methylprednisolone, plasma exchange, and reconstitution of azathioprine and IVIg.

## DISCUSSION

Inflammatory lesions of the spinal cord, whether completely or partially transverse, are referred to as transverse myelitis. Usually, transverse myelitis is an acute illness which develops over several hours and progresses within the next days. Patients typically present with para- or tetra-paresis, depending on the level of spinal cord involvement, and sensory disturbances. Autonomic involvement may present with bowel or bladder dysfunction.

The term longitudinally extensive transverse myelitis (LETM) refers to lesions extending across at least three contiguous vertebral segments. Patients with LETM represent a particular subgroup of transverse myelitis, different from those with shorter lesions; they have a low risk of evolution towards multiple sclerosis, but more severe clinical symptoms.^1^

Although LETM most frequently occurs in association with neuromyelitis optica (NMO), it may be caused by spinal cord infarction, compressive myelopathy, or infectious myelopathy; it could appear in the course of autoimmune or inflammatory conditions such as SLE, sarcoidosis, or Behçet’s disease; rarely, it may be an isolated or idiopathic condition.

Neuromyelitis optica (Devic’s syndrome) is a rare inflammatory neurologic disease, characterized by severe optic neuritis and LETM; it has a relapsing course and is associated with NMO immunoglobulin G (NMO-IgG). These antibodies bind to the water channel aquaporin 4 and are highly specific, occurring in 70%–90% of patients in former NMO series, yet are not found in patients suffering transverse myelitis or optic neuritis as manifestation of systemic autoimmune or rheumatic disease.^2^ The presence of AQP4-IgG provides a possible distinction of NMO from other autoimmune neurologic disorders.

More than 90% of NMO-related LETM patients develop a relapsing–remitting course, accumulating disability; relapse rate is related to the extent of the spinal cord lesion and the presence of AQP4-IgG, but not its titer.^3^ The diverse courses of NMO observed during longitudinal studies led to the broadening of the diagnostic criteria for NMO spectrum disorders (NMOSD). In AQP4-IgG-positive patients, new criteria allow NMOSD diagnosis in patients with clinical or MRI characteristic findings in at least one of six typical CNS regions, including the optic nerve or the spinal cord. An international panel for NMO diagnosis concluded that NMO clinical syndromes in AQP4-IgG-positive patients may coexist with SLE, highlighting that NMOSD is more likely to be co-associated, than a direct complication of active SLE.^4^

The standard of care in NMOSD is based on expert opinion and includes early aggressive immunosuppression inducing remission in an acute episode, followed by maintenance therapy for prevention of relapse. Pulses of i.v. 1 g methylprednisolone for 3–5 consecutive days, followed by oral prednisolone, are the standard of care. Effectiveness of azathioprine and rituximab (an anti-CD20 antibody) was demonstrated in a meta-analysis performed by the NEMOS group^5^ and in recent studies showing reduced relapse rates and disability scores,^6^ leading to the European Federation of Neurological Societies guidelines and the NOMADMUS group of NMO specialists’ recommendations for treatment with azathioprine or rituximab as first-line therapy for relapse prophylaxis, while mycophenolate mofetil, methotrexate, or cyclophosphamide are second-line therapy.^7^

Involvement of the CNS is among the severe complications of SLE, typically presenting with seizures or psychosis, and only rarely with transverse myelitis. However, the American College of Rheumatology classification for neuropsychiatric SLE included all features of NMO as a possible SLE-related CNS manifestation.

Since SLE-related LETM is very rare, data on the clinical course, outcome, and treatment efficacy are largely absent from the literature. The prevalence of SLE-related LETM is higher in young women (77% of patients are women, mean age 30 years) and may be the first presenting SLE feature. Case series noted an association with active extra-neurologic SLE in only 40%–60% of cases.^8^ On the other hand, LETM may occur years after SLE diagnosis, during a flare. Half of LETM patients experience unfavorable neurologic outcome despite aggressive immune suppression, and over a quarter have persistent disability.^9^

The relapse rate of SLE-associated transverse myelitis remains unknown. Iyer et al. observed a monophasic course in 80% of cases, with poor recovery,^10^ but Saison et al. reported common relapses during corticosteroid tapering in 50%–60% of the patients,^8^ confirming the need for maintenance therapy following induction, with some evidence supporting the use of cyclophosphamide, azathioprine, and hydroxychloroquine in this aim.^9^

This therapeutic approach is partly different from the one towards NMO-related LETM; however, it has been suggested by NMO specialists that a more targeted approach may be more effective with fewer adverse effects. Modulation of the complement system in treatment of NMOSD was proposed with eculizumab.^11^ Bevacizumab, an anti-VEGF antibody, was reported as a safe add-on therapy to corticosteroids.^12^ High levels of IL-6 in CNS fluid from patients with NMOSD may justify the use of IL-6 blockade, and clinical trials are ongoing. Aquaporumab, a competitive inhibitor of AQP4, is under investigation.^13^ These innovative treatments have no proven efficacy for treatment of neuropsychiatric lupus, which underscores the importance of correctly diagnosing the etiology of LETM in every patient, particularly in SLE patients.

This case also highlights the long-term efficacy and safety of IVIg as adjuvant therapy for maintenance of remission in MNO-related LETM in our patient. This approach, proposed long ago based on the assumed IVIg mode of action, was recently supported by a retrospective analysis of 20 NMO patients treated with add-on IVIg. In these patients, IVIg treatment was associated with prevention of both relapse and disability progression. Further studies are needed to determine the optimal dose, dosing interval, and efficacy of this treatment.^14^

## SUMMARY

In SLE patients, LETM is more likely to be caused by associated NMOSD than by active neuropsychiatric lupus. The finding of SLE activity in additional sites, immune profiles, and complement or anti-dsDNA levels as well as specific neuro-imaging findings aid to some extent with discriminating these conditions. The presence of AQP4-IgG provides solid grounds for the attribution of LETM as a distinct NMOSD–SLE overlap and is important for relapse prediction and patient education. The treatment of NMOSD, as for SLE-related LETM, should include induction with early aggressive immunosuppression. Maintenance therapy prevents relapse and neurologic disability. Some treatment options are considered appropriate for both NMOSD and SLE-related LETM. Differentiating the two becomes more essential as highly specific and targeted treatment options become available.

In NMO-related LETM, IVIg might be a safe and effective adjuvant therapy for long-term remission maintenance.

## Abbreviations

ANA: antinuclear antibody
APLA: antiphospholipid antibodies
AQP4: anti-aquaporin 4 antibodies
CYC: cyclophosphamide
IgG: immunoglobulin G
LETM: longitudinally extensive transverse myelitis
NMO: neuromyelitis optica
NMOSD: neuromyelitis optica spectrum disorder
SLE: systemic lupus erythematosus

## References

[1] Nardone R, Fitzgerald RT, Bailey A (2015). Longitudinally extensive transverse myelitis in systemic lupus erythematosus: case report and review of the literature. Clin Neurol Neurosurg.

[2] Jarius S, Jacobi C, de Seze J (2011). Frequency and syndrome specificity of antibodies to aquaporin-4 in neurological patients with rheumatic disorders. Mult Scler.

[3] Kessler RA, Mealy MA, Jimenez-Arango JA (2017). Anti-aquaporin-4 titer is not predictive of disease course in neuromyelitis optica spectrum disorder: a multicenter cohort study. Mult Scler Relat Disord.

[4] Wingerchuk DM, Banwell B, Bennett JL (2015). International consensus diagnostic criteria for neuromyelitis optica spectrum disorders. Neurology.

[5] Trebst C, Jarius S, Berthele A (2014). Update on the diagnosis and treatment of neuromyelitis optica: recommendations of the Neuromyelitis Optica Study Group (NEMOS). J Neurol.

[6] Gao F, Chai B, Gu C (2019). Effectiveness of rituximab in neuromyelitis optica: a meta-analysis. BMC Neurol.

[7] Ciron J, Audoin B, Bourre B (2018). Recommendations for the use of rituximab in neuromyelitis optica spectrum disorders. Rev Neurol (Paris).

[8] Saison J, Costedoat-Chalumeau N, Maucort-Boulch D (2015). Systemic lupus erythematosus-associated acute transverse myelitis: manifestations, treatments, outcomes, and prognostic factors in 20 patients. Lupus.

[9] Li XY, Xiao HB, Pai P (2017). Myelitis in systemic lupus erythematosus. J Clin Neurosci.

[10] Iyer A, Elsone L, Appleton R, Jacob A (2014). A review of the current literature and a guide to the early diagnosis of autoimmune disorders associated with neuromyelitis optica. Autoimmunity.

[11] Pilch KS, Spaeth PJ, Yuki N, Wakerley BR (2017). Therapeutic complement inhibition: a promising approach for treatment of neuroimmunological diseases. Expert Rev Neurother.

[12] Mealy MA, Shin K, John G, Levy M (2015). Bevacizumab is safe in acute relapses of neuromyelitis optica. Clin Exp Neuroimmunol.

[13] Bruscolini A, Sacchetti M, La Cava M (2018). Diagnosis and management of neuromyelitis optica spectrum disorders - an update. Autoimmun Rev.

[14] Lim Y-M, Kim H, Lee E-J, Kim HW, Kim HJ, Kim KK (2020). Beneficial effects of intravenous immunoglobulin as an add-on therapy to azathioprine for NMO-IgG-seropositive neuromyelitis optica spectrum disorders. Mult Scler Relat Disord.

